# Supplementary material for: Structural and dynamic insights into supra-physiological activation and allosteric modulation of a muscarinic acetylcholine receptor
Source: Nat Commun. 2023 Jan 23;14:376. doi: 10.1038/s41467-022-35726-z (PMC9870890; doi:10.1038/s41467-022-35726-z)

Supplementary Information for Structural and dynamic insights into supra-physiological activation and allosteric modulation of a muscarinic acetylcholine receptor

Jun Xu *et al.*

a

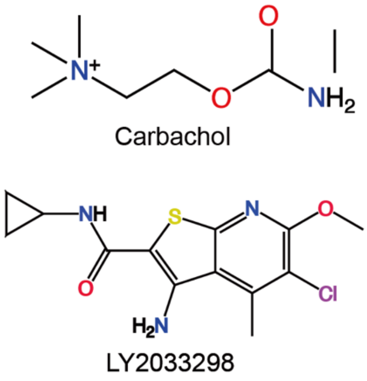

b

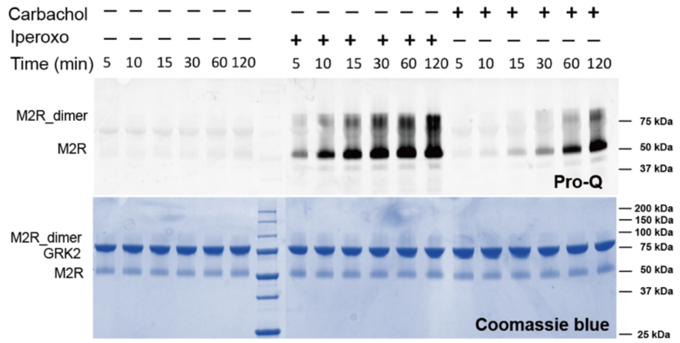

c

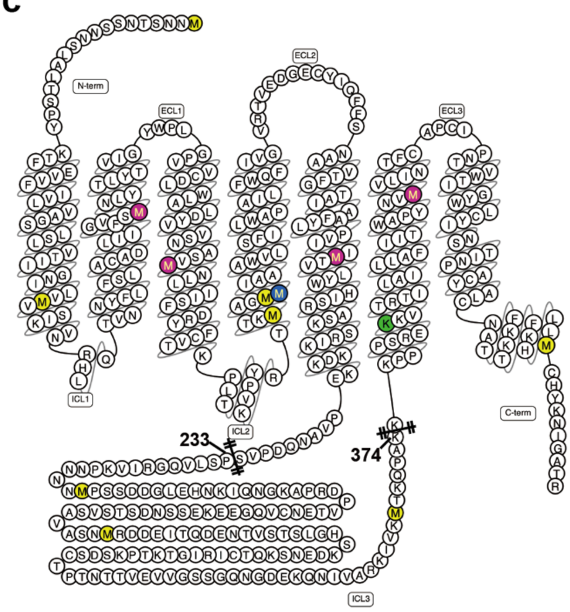

Constructs	Sequence modification/mutations
**M2Rmini**	Truncation of 233-374
**M2Rmini△5M**	Truncation of 233-374<br>Mutation of methionines in yellow
**M2RFL△8M**	Mutation of methionines in yellow
**M2RFL△9M**	Mutation of methionines in yellow and blue
**M2RFL△9M -K383M**	Mutation of methionines in yellow and blue<br>K383 labeled in green

Supplementary Fig. 1: Structures and function of M2R ligands. (**a**) Chemical structures of carbachol and LY2033298. (**b**) SDS-PAGE for the in vitro phosphorylation of the M2RFL△8M by GRK2 in different states and at different times from a single experiment. The same gel was stained by Pro-Q (upper) and Coomassie blue (bottom) sequentially. **(c)** Snake plot of secondary structure and amino acid sequence of the constructs used in this study. Raw picture was adopted from GPCRdb[76].

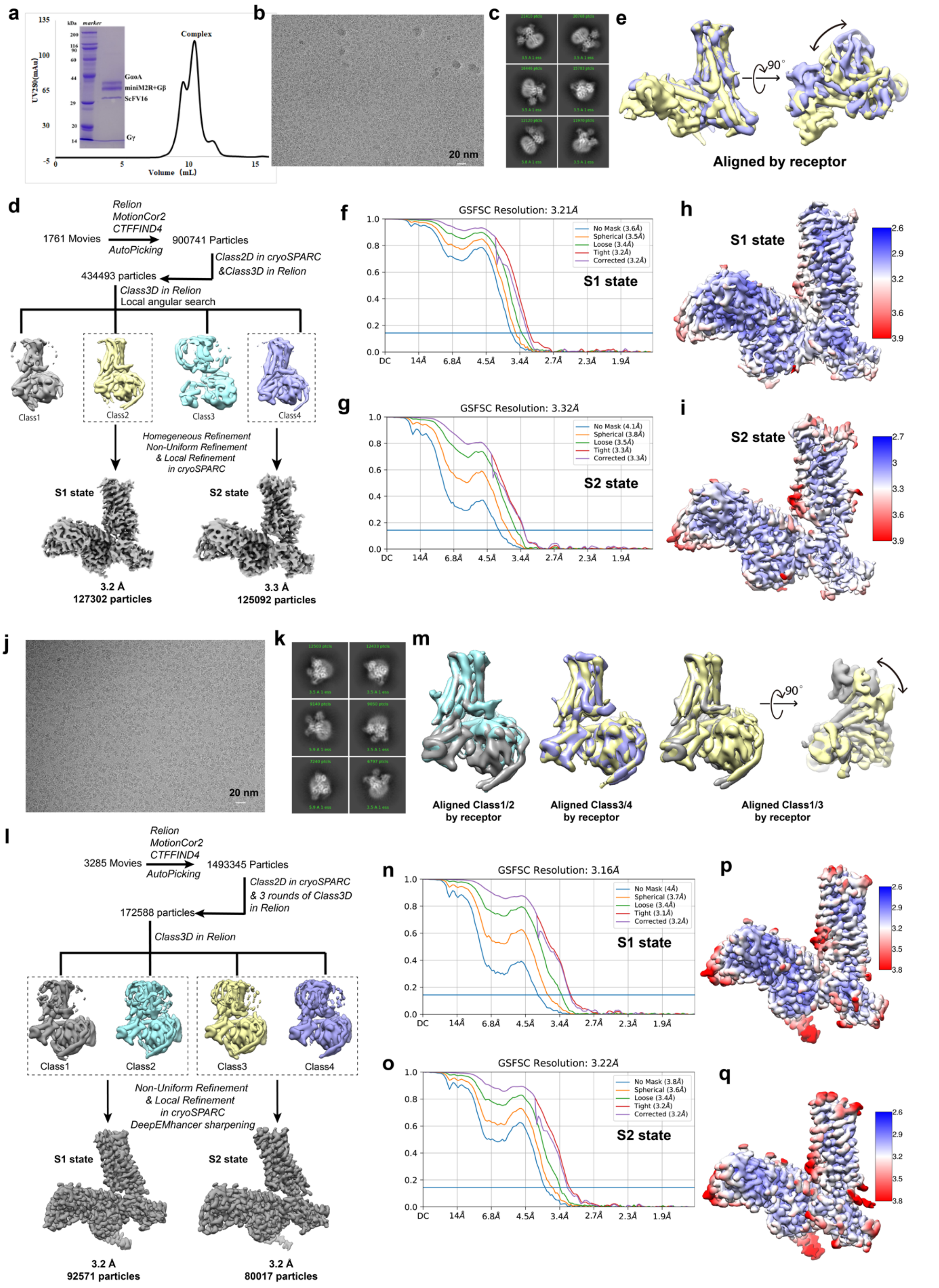

Supplementary Fig. 2: Cryo-EM sample preparation and structure determination. (**a**) Size exclusion chromatography profile and SDS-PAGE of the ACh-M2R-GoA complex from more than three independent experiments. (**b**) Representative micrograph of the ACh-M2R-GoA

complex particles from 1781 micrographs. (**c**) Representative 2D classification results of the ACh-M2R-GoA complex. (**d**) Workflow for image processing of the ACh-M2R-GoA complex. (**e**) Alignment of the 3D classifications in S1 and S2 states based the receptor. The different orientation of GoA is highlighted with black arrow. (**f** and **g**) Fourier shell correlation (FSC) curves with the estimated resolution according to the gold standard. (**h** and **i**) Local resolution maps for ACh-bound S1 (h) and S2 (i) states. (**j**) Representative micrograph of the LY2119620-ACh-M2R-GoA complex particles from 3285 micrographs. (**k**) 2D classification results of the LY2119620-ACh-M2R-GoA complex. (**l**) Workflow for image processing of the LY2119620-ACh-M2R-GoA complex. (**m**) Alignment of different 3D classifications based the receptor. The different orientation of GoA is highlighted with black arrow. (**n** and **o**) Fourier shell correlation (FSC) curves with the estimated resolution according to the gold standard for the LY2119620-bound structures. (**p** and **q**) Local resolution maps for LY2119620-bound S1 (**p**) and S2 (**q**) structures.

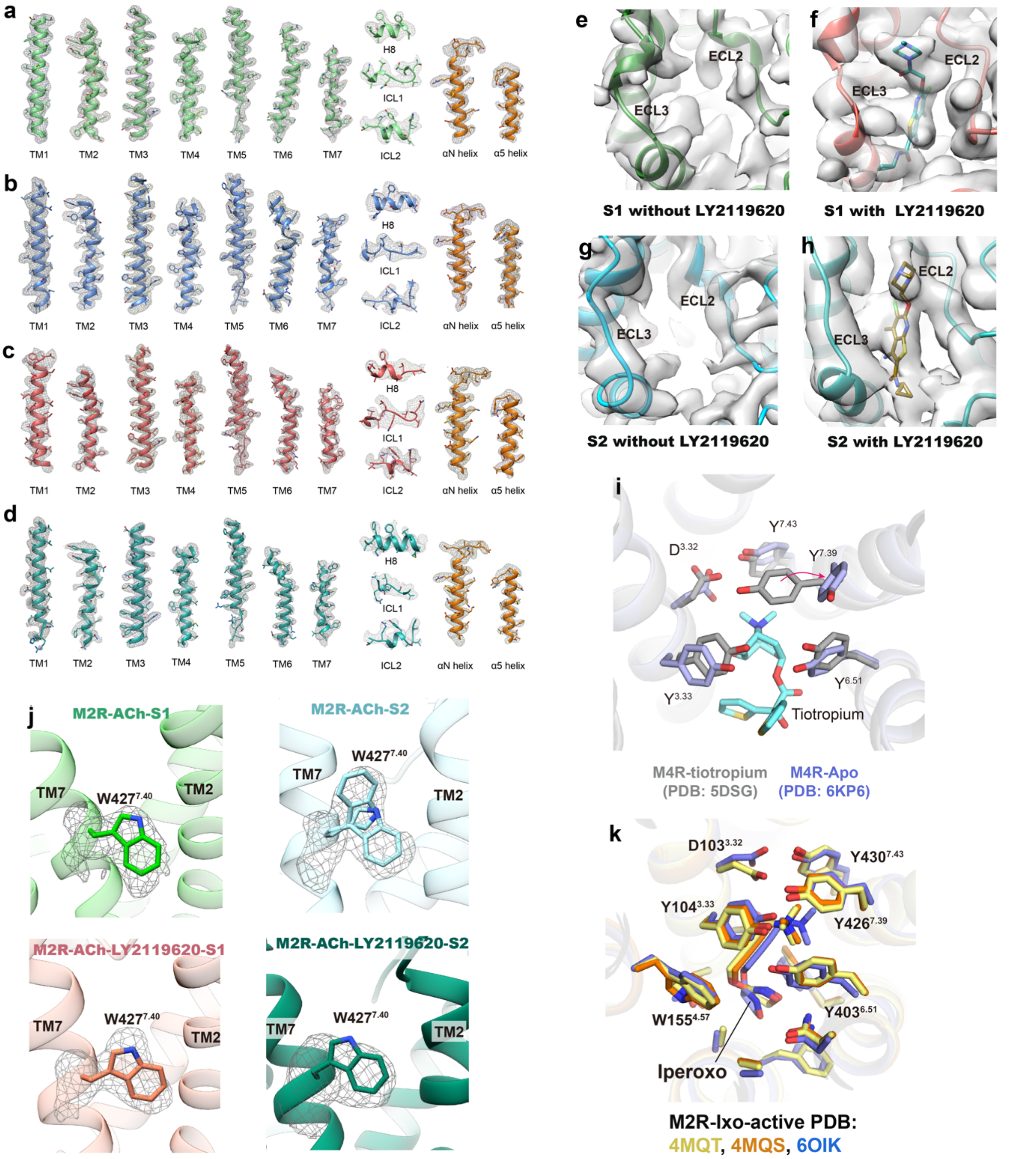

Supplementary Fig. 3: Representative density maps of refined structures and structural comparison of extracellular receptor conformations. (**a-d**) Representative density maps and models for TM1-7, H8, ICL1 and ICL2 of M2R, and the N-terminal and C-terminal α helices of Gαs (αN and α5) of four different structures: (**a**) S1 state of ACh-M2R-GoA, (**b**) S2 state of ACh-M2R-GoA, (**c**) S1 state of LY2119620-ACh-M2R-GoA and (**d**) S2 state of LY2119620-ACh-M2R-GoA. (**e-h**) Comparison of density maps of the extracellular vestibule in different states, S1 without LY2119620 (**e**), S1 with LY2119620 (**f**), S2 without LY2119620 (**g**) and S2 with LY2119620 (**h**). (**i**)Different sidechain conformations of the $Y^{7.39}$ in antagonist-bound (PDB:5DSG) and apo-state (PDB:6KP6) M4R structures. (**j**) Comparison of the sidechain

conformations and density maps of W427$^{7.40}$ in the four complex structures of M2R. The alternative rotamers of W427$^{7.40}$ the S2 state of ACh-M2R-GoA structure are shown. (**k**) Comparison of the orthosteric pocket conformations of Ixo-bound M2R in three different active structures.

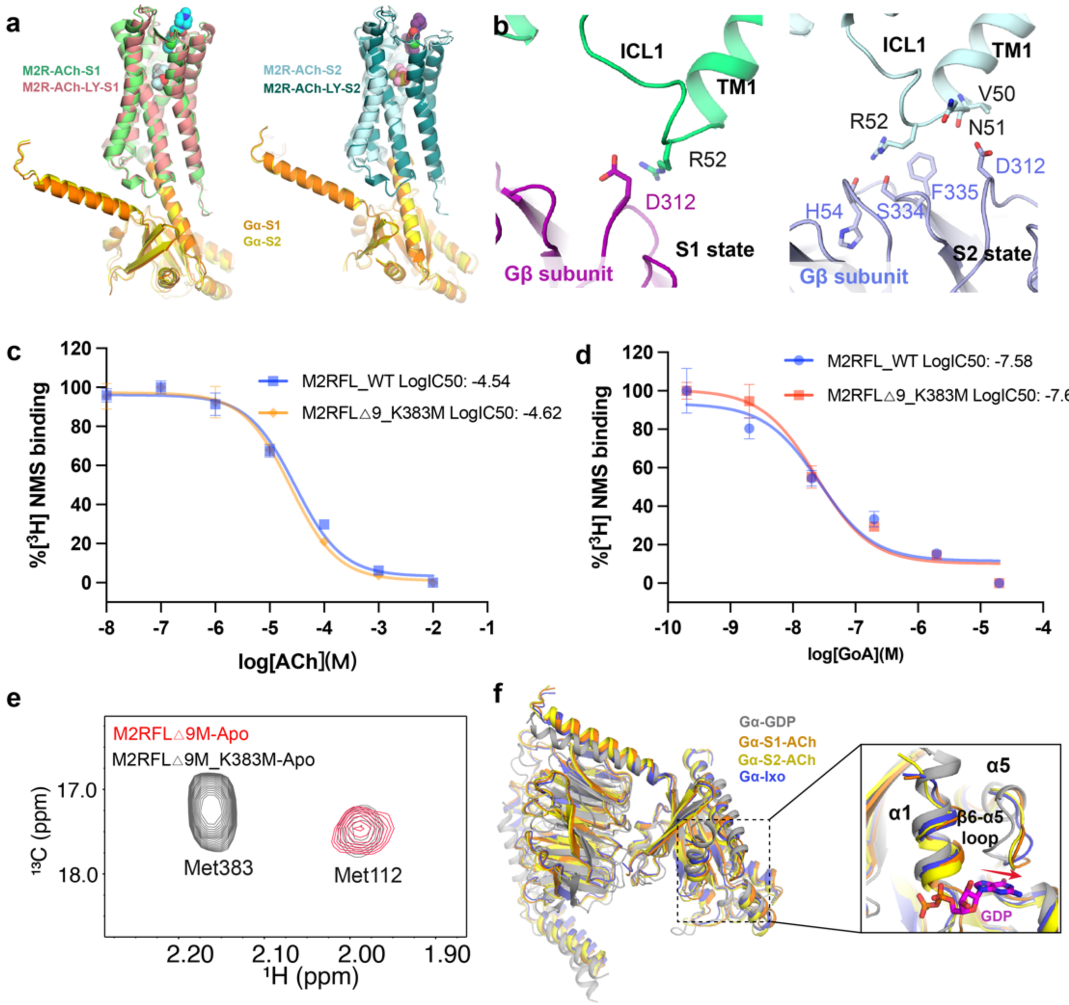

Supplementary Fig. 4: Comparison of ACh and Ixo stabilized M2R-GoA signaling complex. (**a**) Comparison of the GoA coupling orientation of M2R structures in the presence or absence of LY2119620. (**b**) Interactions between of Gβ subunit with ICL1 of M2R in S1 and S2 state. The interface volume in S2 state is larger than S1 state due to the rotation of GoA. Note that the density of R52 in both states are not well-resolved, the detailed interactions maybe slightly different from the present model. (**c**) ACh competition curves show that the ligand property of the engineered construct M2RFL△9M_K383M are similar to that of the wild type full length M2R, referred to as M2RFL_WT. Data are given as mean ± SEM from 3 independent experiments. (**d**) Titration of GoA in the competition ligand-binding assay shows similar GoA coupling for M2RFL△9M_K383M and M2RFL_WT. Coupling of M2R to GoA increases the affinity for ACh, which displaces [^{3}H]-NMS. Data are given as mean ± SEM from 3 independent experiments. (**e**) Assignment of the K383M resonance. (**f**) Alignment of Gαo in four different structures: ACh-M2R-GoA in the S1 and S2 states, Ixo-bound M2R-GoA (PDB: 6OIK) and GDP-bound GoA alone (PDB:1GP2). The enlarged box shows the comparison of conformational differences of β6-α5 loop.

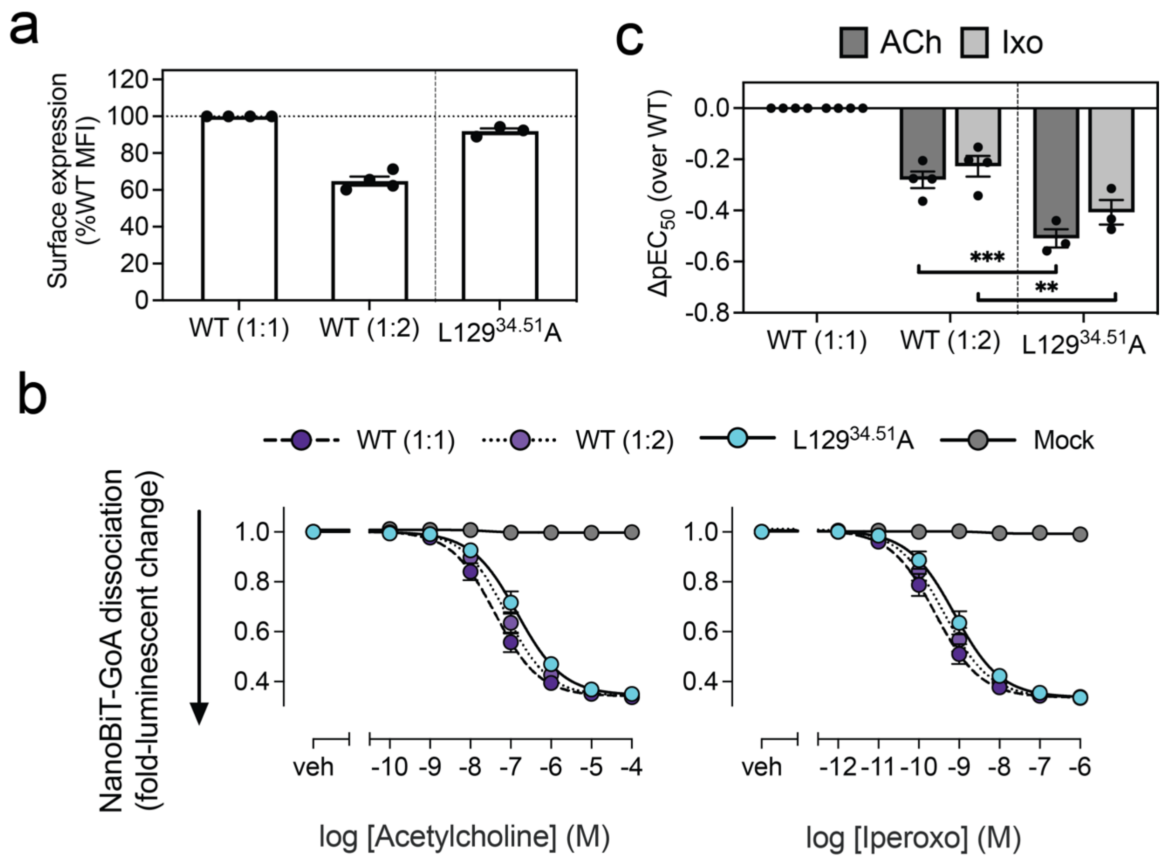

Supplementary Fig. 5: Mutagenesis studies of L129$^{34.51}$A. **(a)** Cell surface expression of WT M2R and the L129$^{34.51}$A mutant were analyzed by flow cytometry. The L129$^{34.51}$A mutant shows an expression level between the same amount (1:1) and the half amount (1:2) of WT M2R plasmid concentrations. Symbols and error bars represent mean and s.e.m. of three or four independent experiments, each performed in duplicate. **(b)** Concentration-response curves for the NanoBiT-GoA dissociation signals for the titrated wild-type M2R and the L129$^{34.51}$A mutant upon ACh or Iperoxo stimulation. Symbols and error bars represent mean and s.e.m. of three or four independent experiments, each performed in duplicate. **(c)** GoA-coupling activity ($\triangle$pEC$_{50}$) were analyzed by the NanoBiT-GoA dissociation assay. Symbols and error bars represent mean and s.e.m. of three or four independent experiments, each performed in duplicate. Statistical analyses were performed using the two-way ANOVA followed by the two-sided Sidak's post-hoc test. **, $p < 0.01$; ***, $P < 0.001$.

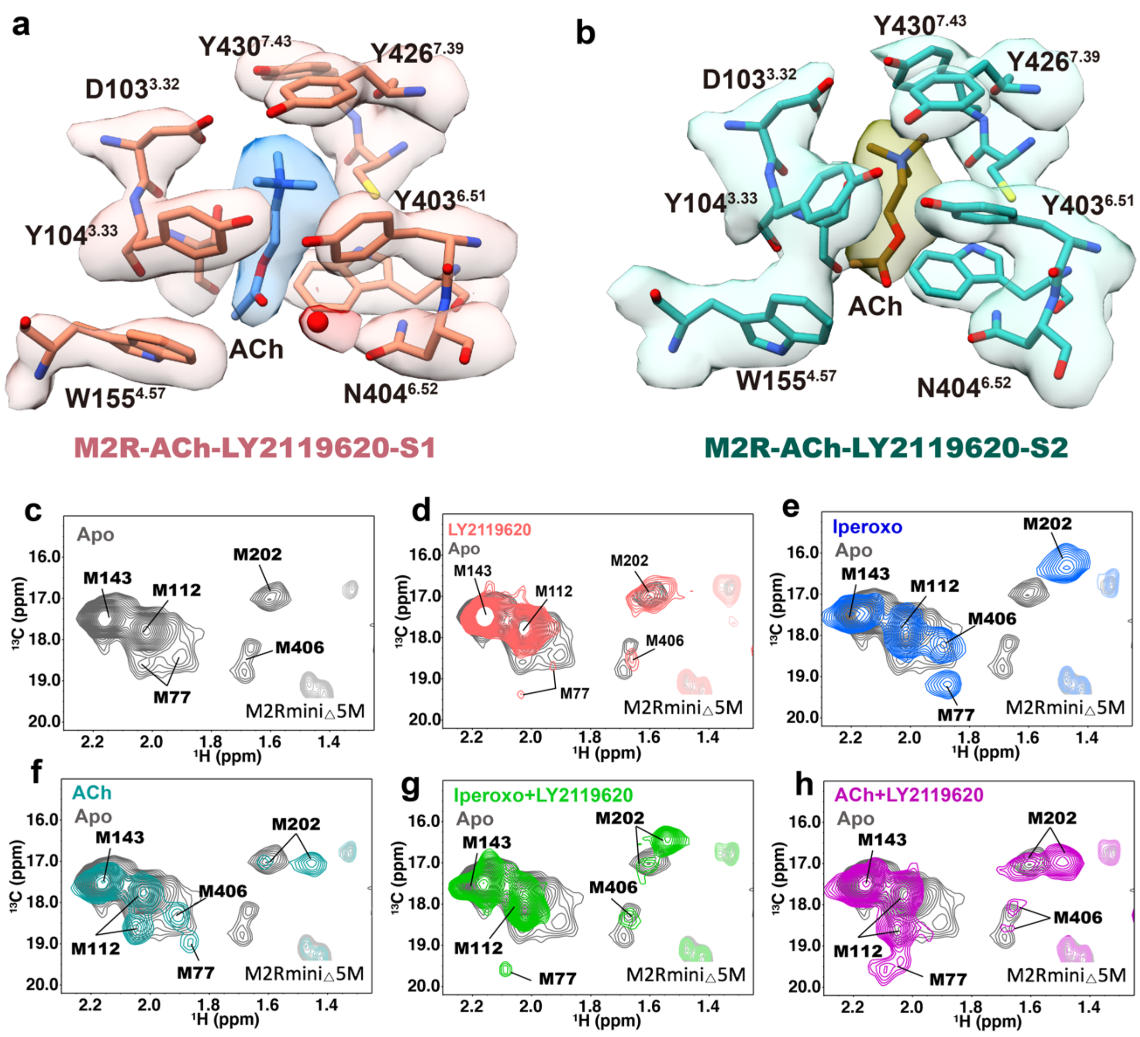

Supplementary Fig. 6: Effect of LY2119620 on the HSQC spectra of M2R. (**a** and **b**) Density maps of residues in the orthosteric pocket in LY2119620-bound S1 (A) and S2 (B) states. The potential water molecule in the LY2119620-bound S1 state is shown as red sphere. (**c**) Spectrum of apo-state M2Rmini△5M. (**d**) Spectrum of LY2119620-bound M2Rmini△5M. (**e** and **f**) Spectra of M2Rmini△5M bound to Ixo (**e**) and ACh (**f**). (**g** and **h**) Spectra of M2Rmini△5M bound to Ixo/LY2119620 (**g**) and ACh/LY2119620 (**h**). The apo-state spectrum is set as reference and shown in gray in all ligand-bound spectra.

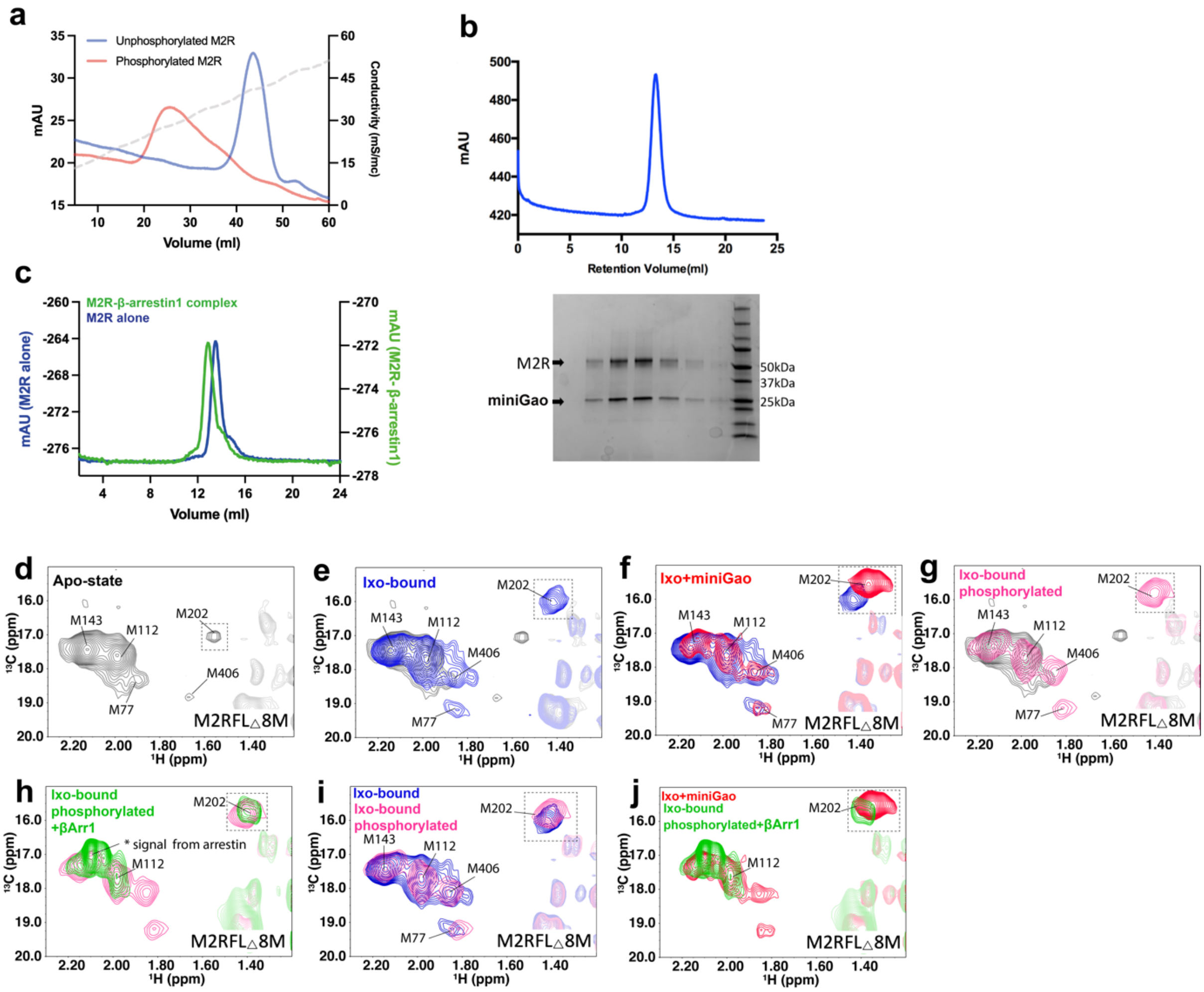

Supplementary Fig. 7: Effect of β-arrestin-1 and miniGαo on the HSQC spectra of M2R. (**a**) Ion-exchange profile of phosphorylated and unphosphorylated M2R. (**b**) Complex formation of the M2RFL△8M with miniGαo from a single experiment. (**c**) Size exclusion chromatography profile of the M2RFL△8M alone (blue) and M2RFL△8M-β-arrestin-1 complex (green). (**d**) Spectrum of apo-state M2RFL△8M shown in gray. (**e**) Overlay of Ixo-bound M2RFL△8M spectrum with apo-state spectrum. (**f**) Overlay of Ixo/miniGαo-bound M2RFL△8M spectrum with Ixo-bound spectrum. (**g**) Overlay of phosphorylated Ixo-bound M2RFL△8M spectrum with apo-state spectrum. (**h**) Overlay of Ixo/β-arrestin-1-bound M2RFL△8M spectrum with the phosphorylated Ixo-bound spectrum. The asterisk indicates natural abundant signals from βArr1, which only appear in the presence of βArr1. (**i**) Comparison of the phosphorylated and unphosphorylated spectra. (**j**) Comparison of the miniGαo and β-arrestin-1 coupled spectra. The spectra of $M202^{5.54}$ is highlighted in dashed black rectangle.

Supplementary Table 1: Potency and maximum efficacy of ACh and Iperoxo in the absence and presence of the modulator LY2119620 at M2R for G-protein activation measured by a cell-based Gqi-inositol phosphate accumulation assay and β-arrestin-2 recruitment determined by the PathHunter assay.

IP accumulation	EC_{50} [nM ± s.e.m.][a]	E_{max} [% ± s.e.m][b]	p-value[c]	n
ACh	240 ± 18	100 ± 0.18	-	14
+ 0.1 µM LY	140 ± 22	97.0 ± 2.4	ns	5
+ 0.3 µM LY	99 ± 12	95.3 ± 1.3	ns	4
+ 1 µM LY	78 ± 22	98.4 ± 1.5	ns	8
+ 3 µM LY	41 ± 17	93.8 ± 2.8	ns	5
+ 10 µM LY	19 ± 3.5	95.6 ± 2.8	ns	7
+ 30 µM LY	18 ± 5.7	92.0 ± 2.8	0.0178	5
+ 100 µM LY	31 ± 7.7	90.5 ± 3.5	0.0081	4
Iperoxo	0.31 ± 0.043	108.6 ± 1.6	-	12
+ 0.1 µM LY	0.13 ± 0.028	109.5 ± 1.9	ns	4
+ 1 µM LY	0.047 ± 0.023	103.2 ± 2.0	ns	6
+ 3 µM LY	0.0099 ± 0.0026	103.9 ± 2.1	ns	8
+ 10 µM LY	0.0089 ± 0.0017	105.5 ± 1.7	ns	8
+ 30 µM LY	0.014 ± 0. 0068	97.3 ± 5.8	0.0125	6
+ 100 µM LY	0.11 ± 0.053	98.9 ± 2.0	0.0274	5
**arrestin recruitment**	EC_{50} [nM ± s.e.m.][a]	E_{max} [% ± s.e.m][b]	p-value[c]	n
ACh	1600 ± 170	100 ± 0.25	-	5
+ 0.1 µM LY	1600 ± 550	99.8 ± 5.3	ns	4
+ 1 µM LY	860 ± 330	119.0 ± 8.3	ns	3
+ 3 µM LY	740 ± 170	135.8 ± 4.9	0.0008	4
+ 10 µM LY	670 ± 370	127.3 ± 10.1	0.0189	3
+ 30 µM LY	1100 ± 270	134.0 ± 5.9	0.0013	4
+ 100 µM LY	2000 ± 580	129.8 ± 6.1	0.0048	4
Iperoxo	0.35 ± 0.080	150.9 ± 2.8	-	11
+ 0.1 µM LY	0.22 ± 0.039	170.5 ± 3.6	0.0148	4
+ 1 µM LY	0.091 ± 0.002	160.3 ± 8.5	ns	4
+ 3 µM LY	0.078 ± 0.008	172.0 ± 1.5	0.0078	4
+ 10 µM LY	0.087 ± 0.020	161.3 ± 8.0	ns	4
+ 30 µM LY	0.12 ± 0.027	168.5 ± 3.1	0.0337	4
+ 100 µM LY	0.20 ± 0.023	164.0 ± 2.4	ns	4

[a] Potency of the agonist in the absence or presence of LY2119620 relative to the full effect of ACh displayed as mean values in % ± s.e.m. derived from 3-14 single experiments (n) each done in duplicates. [b] Maximum efficacy for the agonist in the absence or presence of LY2119620 relative to the full effect of ACh given as mean values in % ± s.e.m.. [c] Significance analyzed by One-way ANOVA applying Dunnett's multiple comparisons test in Prism 8.0 comparing E_{max} of the agonist alone to the E_{max} in presence of a distinct concentration of modulator. Significance threshold was set as 95% confidence interval and displayed as p-value ($p<0.05$).

Supplementary Table 2. Cryo-EM data collection, refinement and validation statistics.

Data collection and processing	ACh-M2R-GoA-scfv16 S1 state<br>EMBD-25748, PDB ID 7T8X	ACh-M2R-GoA-scfv16 S2 state<br>EMBD-25749, PDB ID 7T90	ACh-LY2119620-M2R-GoA-scfv16 S1 state<br>EMBD-25751, PDB ID 7T94	ACh-LY2119620-M2R-GoA-scfv16 S2 state<br>EMBD-25752, PDB ID 7T96
Magnification	105,000	105,000	105,000	105,000
Voltage (kV)	300	300	300	300
Electron exposure (e^{-}/Å)	17.6	17.6	17.5	17.5
Defocus range (μm)	-1.0~-2.0	-1.0~-2.0	-1.0~-2.0	-1.0~-2.0
Pixel size (Å)	0.85	0.85	0.85	0.85
Symmetry imposed	C1	C1	C1	C1
Initial particle images (no.)	900741	900741	1493345	1493345
Final particle images (no.)	127302	125092	92571	80017
Map resolution (Å)	3.21	3.32	3.16	3.22
FSC threshold	0.143	0.143	0.143	0.143
**Refinement**				
Initial model used (PDB code)	6OIK	6OIK	6OIK	6OIK
Map sharpening B-factor (Å)	-133	-82	DeepEMhancer	DeepEMhancer
**Model composition**				
Non-hydrogen atoms	8555	8456	8388	8465
Protein residues	1126	1125	1113	1125
**B factor (Å)**				
Protein	28.5	46.23	37.44	38.44
Ligand	40.02	78.18	79.36	81.88
**R.m.s. deviations**				
Bond lengths (Å)	0.005	0.006	0.005	0.007
Bond angles (°)	1.046	1.175	0.894	0.908
**Validation**				
MolProbity score	1.66	1.86	1.80	1.99
Clashscore	7.42	9.74	7.99	10.39
**Ramachandran plot**				
Favored (%)	96.21	94.94	94.69	92.59
Allowed (%)	3.79	4.97	5.31	7.32
Disallowed (%)	0	0.09	0	0.09

Uncropped scans of all blots and gels

Supplementary Fig. 1b
Pro-Q staining

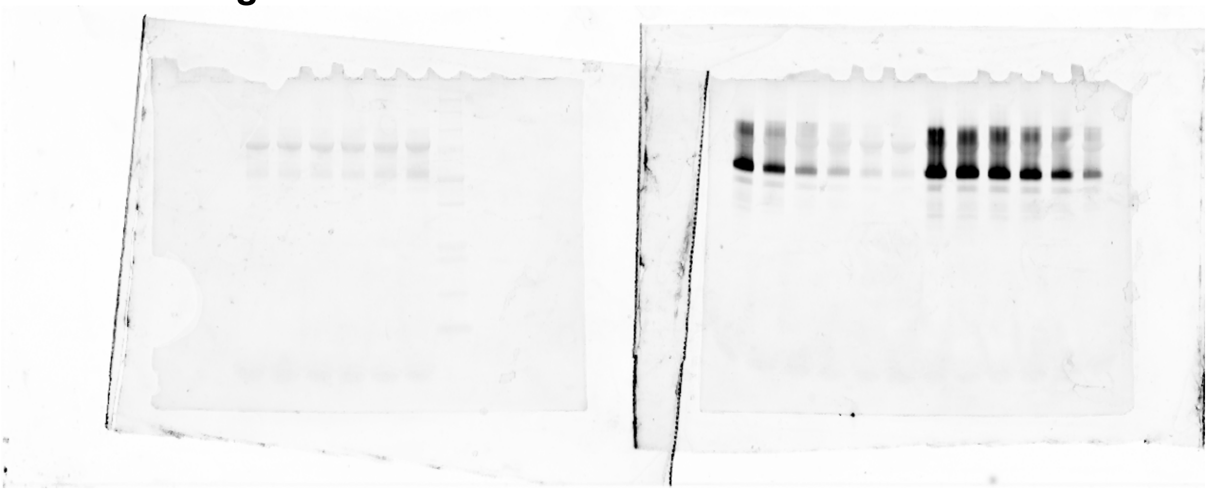

Coomassie blue staining

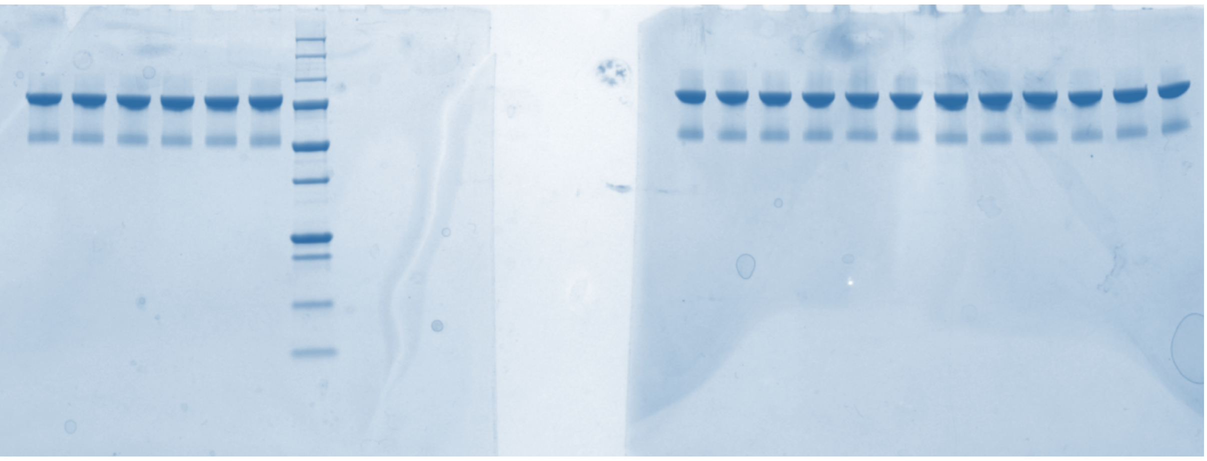

Supplementary Fig. 7b

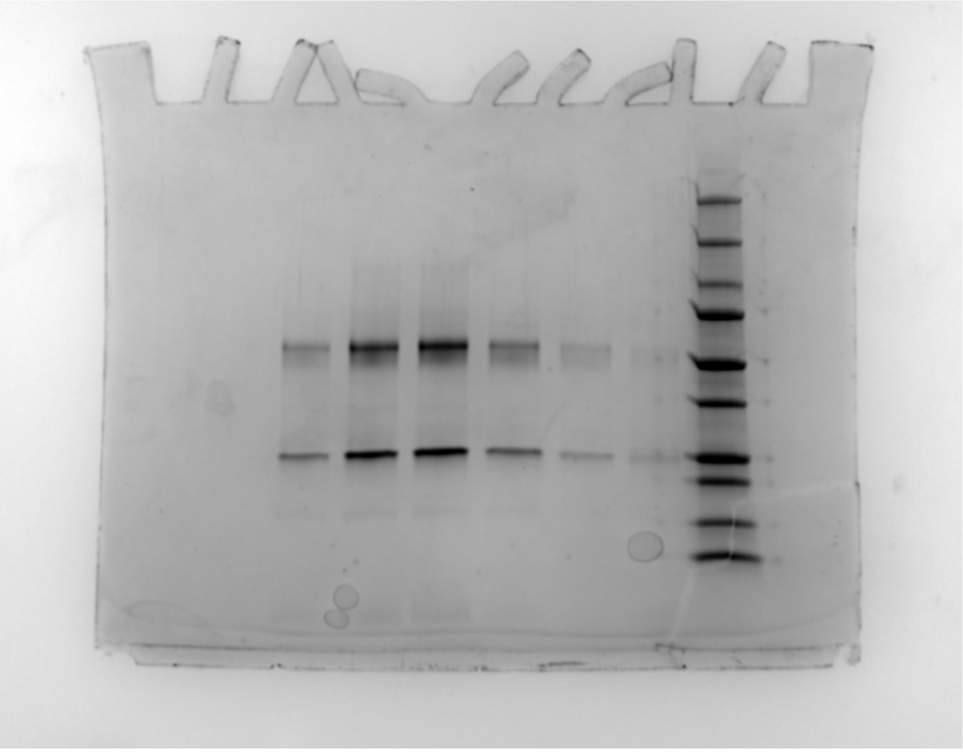

Supplement: Supplementary file 1 — Supplementary Information [file 41467_2022_35726_MOESM1_ESM.pdf]
