# Supplementary material for: Structural and dynamic insights into supra-physiological activation and allosteric modulation of a muscarinic acetylcholine receptor
Source: Nat Commun. 2023 Jan 23;14:376. doi: 10.1038/s41467-022-35726-z (PMC9870890; doi:10.1038/s41467-022-35726-z)
Supplement: Supplementary file 3 — Reporting Summary [file 41467_2022_35726_MOESM3_ESM.pdf]

nature portfolio

Corresponding author(s): Brian K. Kobilka, Changwen Jin, Yang Du

Last updated by author(s): Dec 12, 2022

Reporting Summary

Nature Portfolio wishes to improve the reproducibility of the work that we publish. This form provides structure for consistency and transparency in reporting. For further information on Nature Portfolio policies, see our Editorial Policies and the Editorial Policy Checklist.

Statistics

For all statistical analyses, confirm that the following items are present in the figure legend, table legend, main text, or Methods section.

n/a	Confirmed	
☐	☒	The exact sample size (*n*) for each experimental group/condition, given as a discrete number and unit of measurement
☐	☒	A statement on whether measurements were taken from distinct samples or whether the same sample was measured repeatedly
☐	☒	The statistical test(s) used AND whether they are one- or two-sided<br>*Only common tests should be described solely by name; describe more complex techniques in the Methods section.*
☒	☐	A description of all covariates tested
☐	☒	A description of any assumptions or corrections, such as tests of normality and adjustment for multiple comparisons
☐	☒	A full description of the statistical parameters including central tendency (e.g. means) or other basic estimates (e.g. regression coefficient) AND variation (e.g. standard deviation) or associated estimates of uncertainty (e.g. confidence intervals)
☐	☒	For null hypothesis testing, the test statistic (e.g. *F*, *t*, *r*) with confidence intervals, effect sizes, degrees of freedom and *P* value noted<br>*Give P values as exact values whenever suitable.*
☒	☐	For Bayesian analysis, information on the choice of priors and Markov chain Monte Carlo settings
☒	☐	For hierarchical and complex designs, identification of the appropriate level for tests and full reporting of outcomes
☒	☐	Estimates of effect sizes (e.g. Cohen's *d*, Pearson's *r*), indicating how they were calculated

Our web collection on statistics for biologists contains articles on many of the points above.

Software and code

Policy information about availability of computer code

Data collection	The cryo-EM movie stacks are automatically collected using SerialEM3.7, NMR data are collected using Bruker Topspin Software 4.2.0.
Data analysis	FlowJo 10.7, GraphPad Prism 8.0 and 9.2.0 were used for functional data analysis. MotionCorr2 1.4.0, CTFFIND4, RELION3.0, UCSF Chimera 1.12, Chimera X, CryoSPARC3.2.1, Phenix1.18.2, COOT 0.9.2, MolProbity 4.2 were used for cryo-EM structure determination. NMRPipe/ NMRDraw/NMRViewJ 9.1.0 were used for NMR data processing and analysis.

For manuscripts utilizing custom algorithms or software that are central to the research but not yet described in published literature, software must be made available to editors and reviewers. We strongly encourage code deposition in a community repository (e.g. GitHub). See the Nature Portfolio guidelines for submitting code & software for further information.

Data

Policy information about availability of data

All manuscripts must include a data availability statement. This statement should provide the following information, where applicable:

- Accession codes, unique identifiers, or web links for publicly available datasets
- A description of any restrictions on data availability
- For clinical datasets or third party data, please ensure that the statement adheres to our policy

The Structural models of M1R-GoA, Iperoxo-bound M2R-GoA, The structural data generated in this study have been deposited in the Protein Data Bank (PDB) under

accession codes 7T8X, 7T90, 7T94 and 7T96. The 3D cryo-EM maps have been deposited in the Electron Microscopy Data Bank (EMBD) under accession codes EMDB-25748, EMDB-25749, EMDB-25751 and EMDB-25752. The Structural models of inactive M2R, M1R-G11, Iperoxo-bound active M2R with Nanobody, Iperoxo-bound M2R-GoA, M2R-arrestin, C state and NC state NTS1R-Gi are available in the Protein Data Bank database under the accession codes: 3UON, 6OIJ, 4MQS, 6OIK, 6U1N, 6OS9, 6OSA. The functional and pharmacological data generated in this study are complied in the Source Data file provided with this paper.

Human research participants

Policy information about studies involving human research participants and Sex and Gender in Research.

Reporting on sex and gender	not applicable
Population characteristics	not applicable
Recruitment	not applicable
Ethics oversight	not applicable

Note that full information on the approval of the study protocol must also be provided in the manuscript.

Field-specific reporting

Please select the one below that is the best fit for your research. If you are not sure, read the appropriate sections before making your selection.

☒ Life sciences ☐ Behavioural & social sciences ☐ Ecological, evolutionary & environmental sciences

For a reference copy of the document with all sections, see nature.com/documents/nr-reporting-summary-flat.pdf

Life sciences study design

All studies must disclose on these points even when the disclosure is negative.

Sample size	not applicable
Data exclusions	no data were excluded from analysis
Replication	For cell-based functional assays and in vitro GTPase Glo assay, data from at least 3 independently performed experiments were analyzed and included. All attempts at replication were succeeded.
Randomization	No randomization was attempted or needed. This was not a clinical trial or animal study that is dependent on randomization
Blinding	No blinding was attempted or needed. There was no group allocation performed in this study.

Reporting for specific materials, systems and methods

We require information from authors about some types of materials, experimental systems and methods used in many studies. Here, indicate whether each material, system or method listed is relevant to your study. If you are not sure if a list item applies to your research, read the appropriate section before selecting a response.

Materials & experimental systems

n/a	Involved in the study	
☐	☒	Antibodies
☐	☒	Eukaryotic cell lines
☒	☐	Palaeontology and archaeology
☒	☐	Animals and other organisms
☒	☐	Clinical data
☒	☐	Dual use research of concern

Methods

n/a	Involved in the study	
☒	☐	ChIP-seq
☒	☐	Flow cytometry
☒	☐	MRI-based neuroimaging

Antibodies

Antibodies used	anti-DYKDDDDK mouse monoclonal antibody (Wako Pure Chemicals, Clone 1E6, catalog no. 018-22381, https://labchem-wako.fujifilm.com/europe/product/detail/W01W0101-2238.html), goat anti-mouse IgG secondary antibody conjugated with Alexa

	Fluor 488 (Thermo Fisher Scientific, catalog no. A32723, https://www.thermofisher.com/antibody/product/Goat-anti-Mouse-IgG-H-L-Highly-Cross-Adsorbed-Secondary-Antibody-Polyclonal/A32723).
Validation	The purchased antibodies were well validated by the manufactures in their specific data sheets.

Eukaryotic cell lines

Policy information about cell lines and Sex and Gender in Research

Cell line source(s)	Sf9 cells (Expression Systems, Cat 94-001F); Highfive cells (Expression Systems, Cat 94-002F), HEK-293T (ATCC CRL-3216)
Authentication	Cells lines are maintained by the supplier. No additional authentication was performed by the authors of this study
Mycoplasma contamination	No mycoplasma contamination has been found in the cell lines used in this study.
Commonly misidentified lines (See ICLAC register)	None used
